# Characterisation of Connexin Expression and Electrophysiological Properties in Stable Clones of the HL-1 Myocyte Cell Line

**DOI:** 10.1371/journal.pone.0090266

**Published:** 2014-02-28

**Authors:** Priyanthi Dias, Thomas Desplantez, Majd A. El-Harasis, Rasheda A. Chowdhury, Nina D. Ullrich, Alberto Cabestrero de Diego, Nicholas S. Peters, Nicholas J. Severs, Kenneth T. MacLeod, Emmanuel Dupont

**Affiliations:** Myocardial Function Section, National Heart and Lung Institute, Imperial College London, London, United Kingdom; Dalhousie University, Canada

## Abstract

The HL-1 atrial line contains cells blocked at various developmental stages. To obtain homogeneous sub-clones and correlate changes in gene expression with functional alterations, individual clones were obtained and characterised for parameters involved in conduction and excitation-contraction coupling. Northern blots for mRNAs coding for connexins 40, 43 and 45 and calcium handling proteins (sodium/calcium exchanger, L- and T-type calcium channels, ryanodine receptor 2 and sarco-endoplasmic reticulum calcium ATPase 2) were performed. Connexin expression was further characterised by western blots and immunofluorescence. Inward currents were characterised by voltage clamp and conduction velocities measured using microelectrode arrays. The HL-1 clones had similar sodium and calcium inward currents with the exception of clone 2 which had a significantly smaller calcium current density. All the clones displayed homogenous propagation of electrical activity across the monolayer correlating with the levels of connexin expression. Conduction velocities were also more sensitive to inhibition of junctional coupling by carbenoxolone (∼80%) compared to inhibition of the sodium current by lidocaine (∼20%). Electrical coupling by gap junctions was the major determinant of conduction velocities in HL-1 cell lines. In summary we have isolated homogenous and stable HL-1 clones that display characteristics distinct from the heterogeneous properties of the original cell line.

## Introduction

Cardiac structure and function are most commonly studied using primary cultures of neonatal and adult cardiac myocytes. However their inability to divide and retain their differentiated phenotype in culture limits their use. The development of the HL-1 cell line derived from a mouse atrial myocyte tumour overcomes this particular difficulty [4],[14]. HL-1 cells share similar characteristics with primary cultures of cardiac myocytes, such as general ultrastructural features, cytoplasmic organisation and myofibrillogenesis. They also express a number of cardiomyocyte markers such as α-myosin heavy chain, desmin and connexin 43 (Cx43) [4]. In addition, electrophysiological studies on the HL-1 cells have identified the functional expression of several ion channels such as the L- and T-type calcium (Ca^2+^) channels and the hyperpolarization-activated cyclic nucleotide-gated ‘pacemaking’ channel [1],[29],[32],[42]. The ability of HL-1 cells to proliferate while maintaining a cardiac phenotype in culture allows the use of specific molecular tools such as RNA interference thereby making them a useful cell model to study some aspects of cardiac physiology [41].

One problem with using the HL-1 cell line is that studies have shown the cells to be functionally heterogeneous. For example, Sartiani et al. [32] reported the presence of hyperpolarisation-activated *I*_f_ current in only 30% of the cells patched together with action potentials of different characteristics. Some studies have taken advantage of this cellular heterogeneity. In a study on mitochondrial function during ischemic preconditioning, Pelloux et al. [29] selected cells that were non-contractile to identify any morphological changes that were taking place. However, because in future work we wish to focus on the functional consequences of variations in protein expression, it was essential to obtain a homogeneous cell line thereby excluding any differences due to cellular heterogeneity of the original cell line. To obtain homogeneous cell lines, colonies were selected from low density HL-1 cultures that were visually contracting and showed evidence of electrical automaticity and functional cellular Ca^2+^ regulatory systems. As a result of this process five clones were generated. The aim of the work detailed in this paper was to characterise the (1) homogeneity of the clones and (2) functional determinants considered to be associated with action potential propagation, namely the sodium (Na^+^) channels, the Ca^2+^ handling proteins, and gap junctions at both the molecular and physiological level.

## Materials and Methods

### Sub-cloning

The HL-I cells were obtained from Dr W. C. Claycomb (Louisiana State University Health Centre, New Orleans, LA, USA) [4]. To obtain homogenous cells lines, the original HL-1 cells were split at low density (1∶250 to 1∶500) into 100 mm dishes. Although majority of the cells never divided, some colonies could be visualised contracting after 2–3 weeks in culture. Further microscopical examination revealed clusters of cells that were synchronously contracting (see results for further details). These groups of cells were isolated using cloning cylinders, seeded into 24 well plates and split 1∶3 to 1∶4 after reaching confluency.

### Cell culture

HL-1 clones were cultured under a atmosphere of 5% CO_2_ and 95% air at 37°C in Claycomb medium supplemented with 10% foetal bovine serum, 4 mm L-glutamine and 100 µM norepinephrine as previously described [4]. Cultures were grown to high density and then split 1∶3 to 1∶4 every five to six days when full confluency was reached to ensure the clones retained their differentiated characteristics. The medium was changed every 24–48 hours. Cells were split by adding trypsin-EDTA to the culture dishes for 5–10 minutes. Trypsin activity was blocked by trypsin inhibitor from glycine max (soya bean) at a ratio of 10 µl per 1 cm^2^ of cells. The dissociated cells were then plated for a new passage and used for their respective studies (e.g. voltage clamp, microelectrode arrays (MEAs) and immunofluorescence).

To verify the functional stability of the HL-1 clones over time, repeated experimentation (e.g. MEAs and patch clamp electrical recordings) was performed at different passages (from passages 10–25).

### Northern blot analysis

To obtain probes for northern blots, plasmids containing partial or complete coding sequences were cut with appropriate endonucleases and purified by low melt agarose gel electrophoresis in TAE buffer (40 mM Tris-acetate, pH 8, 1 mM EDTA). As a probe for the Na^+^/Ca^2+^ exchanger (NCX1), we used the Kpn1 to Apa1 fragment (mouse SLC8A1, 229 bp, a gift from D. Nicoll, UCLA, USA). For the L-type α_1C_ subunit, we used the EcoR1 to EcoRV fragment of the rabbit cDNA (CACNA1C, 2136 bp, a gift from P. Vangheluwe, KU Leuven, Belgium). For the T-type α_1H_ subunit we used the EcoR1 fragment of the human cDNA (CACNA1H, 2269 bp, a gift from M. Shattock, Kings College London, UK). For the cardiac ryanodine receptor (RyR2), we used an EcoR1 to EcoRV fragment (human RyR2, 972 bp, a gift from C. George, Cardiff University). For the sarco-endoplasmic reticulum Ca^2+^ ATPase 2 (SERCA2), we used an Apa1 fragment (human ATP2A2, 1557bp, a gift from P. Vangheluwe, KU Leuven, Belgium). For Cx40, Cx43 and Cx45, we used mouse sequences (gifts from K. Willecke, University of Bonn, Germany) cut respectively with Pst1 (950 bp), EcoR1 to EcoRV (812 bp) and Pst1 (1081 bp).

Total cellular RNA was purified from confluent monolayers of HL-1 clones using a modified guanidinium isothiocyanate/acid phenol extraction procedure [30]. Equal amounts (5 µg/lane) of each sample were run in formaldehyde agarose gels and capillary-transferred onto Hybond N nylon membranes (Amersham). High stringency hybridization was performed at 65°C in 7% SDS, 0.5 M Na^+^ phosphate, pH 7.4 using random-primed probes (Kit supplied by GE Healthcare) generated from gel-purified DNA fragments. The RNA samples were stained with ethidium bromide to visualise the 18S and 28S ribosomal RNA bands, check the equivalent loading of samples and measure the molecular mass of the bands obtained.

### Quantification of connexin expression by western blotting

For western blotting, cells were washed 2 to 3 times with PBS and lysed in a solution containing 20% SDS (10 µl/cm^2^ of confluent HL-1 cells) [5]. Cell lysates (1 µg/lane for Cx40 detection, 0.5 µg/lane for Cx43 detection) were run on 12.5% SDS polyacrylamide gels and electrophoretically transferred to polyvinylidene fluoride membranes (Immobilon-P). The resulting replicas were incubated with the anti-connexin antibodies (Cx43: Sigma C-6219; Cx40: Santa Cruz SC-20466) followed by the appropriate alkaline phosphatase-conjugated secondary antibodies (donkey anti-rabbit IgG for anti-Cx43 and rabbit anti-goat IgG for anti-Cx40). The enzymatic activity was revealed using 5-bromo-4-chloro-3-indolyl-phosphate/nitro blue tetrazolium substrate (BCIP/NBT) solution (Promega). Quantification of western blots was done with densitometric scanning. Linearity of optical density was verified by loading a range of total protein amounts and scanning the resulting immunolabelled membrane.

### Immunofluorescence confocal microscopy

Immunoconfocal microscopy was used to analyse the expression patterns of cardiac sarcomeric skeletal protein α-actinin and gap junctional proteins Cx40, Cx43 and Cx45 in the HL-1 clones. Cells cultured on coverglass were fixed with ice-cold methanol, and immunolabelled. The primary antibodies used were a mouse monoclonal anti-α-actinin (Sigma A-7811), our custom-designed guinea pig polyclonal anti-Cx45 ((Q14E) GP42) [5], a rabbit polyclonal anti-Cx40 ((S15C) R83) [5] and a mouse monoclonal anti-Cx43 (Chemicon MAB-3068). For single labelling appropriate secondary antibodies labelled with Cy3 were used. For the triple labelling Cx40/Cx43/Cx45, the anti-Cx45 was applied first, followed by the anti-Cx43 and then the anti-Cx40. For the secondary antibodies the anti-guinea pig labelled with Cy3 was applied first, then the anti-mouse labelled with FITC followed by the anti-rabbit labelled with Cy5. Negative controls included (1) omission of the primary antibody and (2) using each primary antibody with matching and non-matching secondary antibodies for multiple labelling. All secondary antibodies were confirmed to be species specific for their individual primary antibody. Confocal laser scanning microscopy of immunolabelled sections was carried out using a Leica TCS SP equipped for the detection of Cy3, FITC and Cy5 fluorescence. The images were recorded by single- or sequential dual/triple-channel scanning and are presented as projection views that encompass the full thickness of the cells.

### Imaging of calcium transients

Intracellular Ca^2+^ transients were visualized with the Ca^2+^-sensitive fluorescent dye fluo-4 AM (Molecular Probes, Invitrogen). Cells grown in 35 mm glass bottomed dishes (MatTek Corporation) were loaded with fluo-4 AM (10 µM) and incubated in an atmosphere of 5% CO_2_ and 95% at 37°C for 20 minutes in Claycomb medium. The dishes were then washed to remove excess fluo-4 AM, allowed to de-esterify for 20 minutes and placed on the stage of a confocal laser scanning microscope (Bio-Rad Radiance 2000) for line scanning. The same dye-loaded preparations were scanned in 2-dimensions using an Axio Observer Inverted Widefield Microscope to study the effects of carbenoxolone and lidocaine. During the recordings, the cell preparations were superfused with normal Tyrode at 37°C and fluorescence measured across groups of cells using line scans at an excitation wavelength of 488 nm and a recording wavelength of 520 nm. Fluorescence changes were normalised to the background (resting) fluorescence (F/F_0_). Offline analysis of these recordings were done using ImageJ software.

### Single and dual voltage patch clamp studies

Cells were seeded on glass cover slips and placed in a perfusion chamber of an inverted microscope (Nikon, Diaphot) and superfused at room temperature (23°C) in a modified Krebs-Ringer solution containing (in mmol/L): 140 NaCl, 4 KCl, 2 CaCl_2_, 1 MgCl_2_, 5 HEPES (pH 7.4) 5 glucose, 2 Na-pyruvate. Patch pipettes were filled with internal solution containing (in mmol/L): 130 K-aspartate, 10 NaCl, 1 CaCl_2_, 10 EGTA (free Ca^2+^: pCa 8.1 or ∼7.6 nM), 3 MgATP, 5 HEPES (pH 7.2). For whole cell recordings, pipettes were mounted on a micromanipulator and connected to an amplifier (Multiclamp 700A, Axon Instruments). Signals were filtered (Bessel filtering) and digitized (10 kHz) before data acquisition. Clampfit software (Axon Instruments) was used for data analysis; GraphPad Prism and SigmaPlot were used for curve fitting and statistics.

Current clamp experiments in the whole cell configuration were performed on single cells to record the resting membrane potential (*V*_rp_) and action potentials. Depolarising current pulses from a holding current (*I*_h_) of 0 pA to 40 pA, in 5 pA increments with a 2 ms duration were applied. Voltage clamp mode was applied to record the Na^+^ (*I*_Na_) and Ca^2+^ currents (*I*_Ca_). *I*_Na_ was recorded in the whole cell configuration from a holding potential (*V*_h_) of −80 mV to 50 mV in 10 mV increments and 100 ms in duration. *I*_Ca_ was recorded by applying a conditioning pulse from *V*_h_ of −80 mV to −40 mV for 200 ms to fully inactivate *I*_Na_, followed by depolarizing pulses from *V*_h_ of −40 mV to 50 mV in 10 mV increments and 250 ms in duration. The currents were normalized to the cell membrane capacitance (*C*_m_) and averaged. The values were not corrected for the junction potential (pipette offset), this was compensated prior to giga-seal formation.

Experiments on cell pairs were performed by applying the dual voltage clamp method in the whole cell configuration to determine the macroscopic gap-junctional conductance *g*_j,0_ as previously described [7]. In brief, initially, both cell membrane potentials were clamped at *V*_m1_ = *V*_m2_ = 0 mV to prevent the interference of any non-junctional membrane current. Thereafter, a junctional potential *V*_j_ = *V*_m2_−*V*_m1_ = 10 mV was applied and the gap-junctional current (*I*_j_) was recorded and measured. The junctional conductance *g*_j,0_ was calculated as *g*_j,0_ =  *I*_j_ / *V*_j_. Values of gap junction coupling were corrected for series resistances [39].

### Microelectrode array recordings and analysis

The MEA plates (Multi Channel Systems, Reutlingen, Germany) consist of 60 titanium nitride electrodes (diameter = 30 µM; inter-electrode distance = 200 µm) in an 8×8 grid fabricated on a glass substrate. Cells were seeded as a drop directly on top of the electrodes which were pre-coated with fibronectin (10 mg/ml in water), left to attach for 20 minutes, and the array flooded with Claycomb medium. Recordings were made 2–4 days post-seeding in Claycomb medium at 37°C. Cells were stimulated by applying bipolar voltage pulses (10 ms in duration, 500 to 1000 mV in amplitude and a frequency of 1 to 2 Hz) using a row of electrodes located at the edge of the array. The extracellular field potentials were recorded by each electrode [19],[37],[44], and acquired at a sampling frequency of 25 kHz. The CV was calculated as:
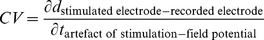




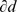
 represents the distance (µm) between the stimulating electrode and the recorded electrode and 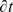 is the time delay (ms) between the artefact of stimulation and the corresponding field potential. The effects of the anti-arrhythmic drugs lidocaine, a Na^+^ channel blocker and carbenoxolone, a gap junction uncoupler were also examined using MEAs. A dose-response curve for each drug was determined in clones 3 and 6, in addition to providing information on CV, also determined suitable drug doses for optical mapping. The drugs were prepared in normal Tyrode solution. Analysis of extracellular recordings made using MC-Rack (Multichannel Systems) was done offline using Excel and GraphPad Prism. At least 10 electrograms from 16 electrodes were analysed for each preparation.

### Statistical analysis

Statistical analyses were done using GraphPad Prism 4 (GrapPad Software Inc., San Diego, California, USA). Data are expressed as a mean ± SEM. It should be noted that the mean data includes experiments recorded over several passages to verify the stability of the clones in long-term culture. All data were compared statistically using a one-way ANOVA followed by a Bonferroni test unless otherwise stated. Statistical differences were judged significant when p<0.05.

## Results

### Spontaneous contractile activity of HL-1 clones

Of the five clones obtained, labelled 1, 2, 3, 4 and 6, clone 1 lost its ability to contract and display electrical activity after several passages, clone 2 stopped contracting immediately upon passaging and clones 3, 4 and 6 maintained a stable contractile phenotype. The appearance of spontaneous contraction was dependent upon the time in culture. Cells in clone 6 displayed spontaneous contraction 24 hours post-seeding despite their low density, while clone 3 and 4 required growth to almost 60–70% confluency to produce spontaneous contractions 3–4 days post-seeding.

### Molecular characterisation of the HL-1 clones

**Figure 1:**
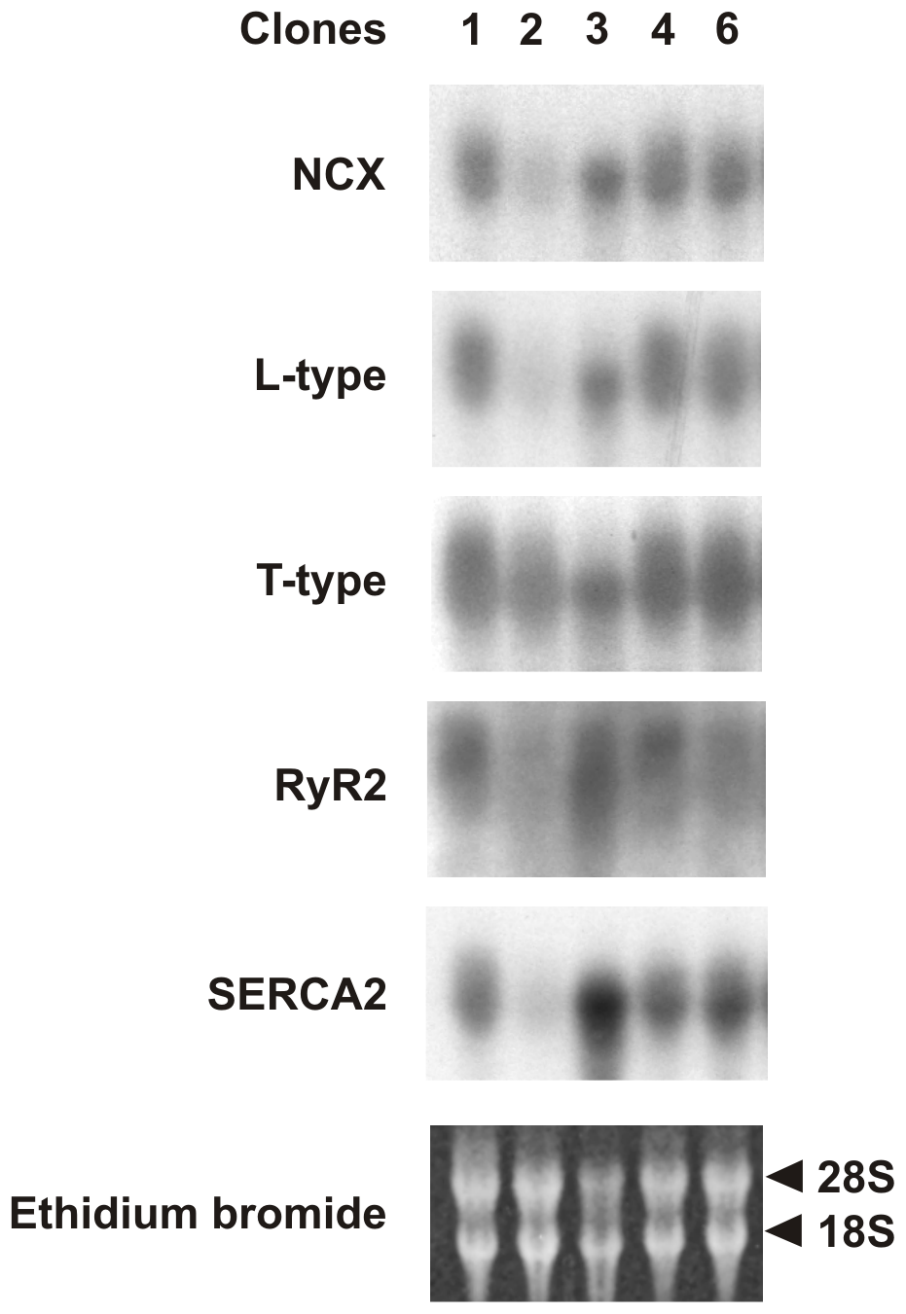
Northern blot analysis of total RNA extracted from the HL-1 clones (indicated on top of the picture). Membranes were hybridised with probes for NCX, L-type Ca^2+^ channel, T-type Ca^2+^ channel, RyR2 and SERCA2 as indicated on the left of the picture. Membranes were stained with ethidium bromide to visualize the ribosomal RNA bands 18S and 28S.

Northern blot experiments were carried out to determine the expression of mRNA coding for the Ca^2+^ handling proteins in all five clones (Figure 1). All the probes labelled a single mRNA band of the expected size. Different exposure times were required to obtain similar signal intensities but this also helped to estimate the relative levels of transcript expressed in the HL-1 clones (Table 1). With the exception of the α_1C_ subunit of the L-type Ca^2+^ channel levels of mRNA expression for NCX, α_1H_ subunit of the T-type Ca^2+^, RyR2 and SERCA2 were similar in clones 1, 2, 3, 4 and 6. In clone 2, transcripts of the L-type Ca^2+^ channel were expressed at significantly lower levels in comparison to the other clones (Table 1).

**Table 1:** Summary of the transcript analysis.

Probe	Size of band	1 (%)	2 (%)	3 (%)	4 (%)	6 (%)
NCX	∼7 kB	88±9	42±21	87±13	94±15	100
L-type	∼8 kB	92±5	50±9^***^	94±5	107±1	100
T-type	∼5 kB	77±4	78±8	87±6	99±5	100
RyR2	∼16 kB	116±16	100±3	128±6	145±27	100
SERCA2	∼4 kB	144±32	33±9	121±4	81±6	100
Cx40	∼3 kB	85±3	11±3^**^	67±11	72±22	100
Cx43	∼3 kB	80±7	16±10^***^	74±4	66±4*	100
Cx45	∼2 kB	90±2	39±4^**^	24±10^**^	94±9	100

* mRNA expression levels were compared with clone 6.

**Figure 2:**
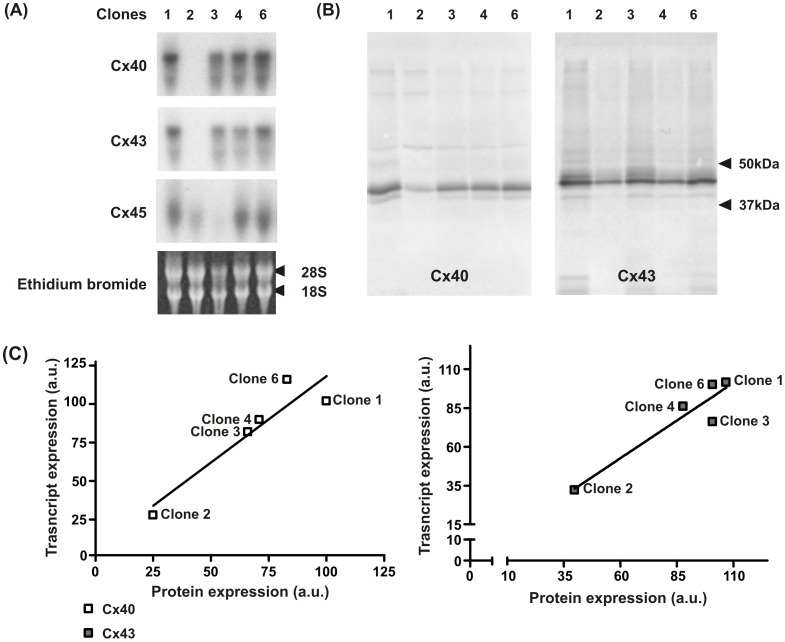
Northern and Western analysis of connexin expression in the HL-1 clones. (A) Membranes were hybridised with probes for Cx40, Cx43 and Cx45 as indicated on the left of the picture. The three connexins were expressed at varying levels by all the HL-1 clones. Membranes were stained with ethidium bromide to visualize the ribosomal RNA bands 18S and 28S. (B) Western blot analysis of Cx40 and Cx43 (B). The Cx40 antibody detected a single band migrating at 40 kDa expressed at similar levels in all the HL-1 clones except clone 2. Cx43 analysis obtained a single band migrating at 43 kDa. As with Cx40, Cx43 was present at similar levels in all HL-1 clones with the exception of clone 2. The clone numbers for transcript analysis and protein expression are indicted at the top of the picture. (C) There is a positive correlation of connexin transcript to protein expression for Cx40 and Cx43.

Transcripts for Cx40, Cx43 and Cx45 (Figure 2 (A)) were present in the HL-1 clones. With the exception of clone 2, Cx43 and Cx40 mRNA levels were similar in the HL-1 clones. Cx45 mRNA was expressed at significantly lower levels in clones 2 and 3 compared with clones 1, 4 and 6. Cx40 and Cx43 mRNA levels were also 3 to 4 times lower in clone 2 compared with any other (Table 1). We assessed connexin expression at the transcriptional or translational stage. Cx40 and Cx43 protein levels were measured (Figure 2 (B) and compared to the corresponding mRNA transcribed in the HL-1 clones (Figure 2 (C)). Western blots for Cx40 and Cx43 revealed a comparable pattern of expression to the corresponding mRNA in all three clones. Cx45 remained undetectable with our western blot protocol indicating that Cx45 expression levels are at least twenty times lower than Cx40 or Cx43.

### Morphological characterisation

**Figure 3:**
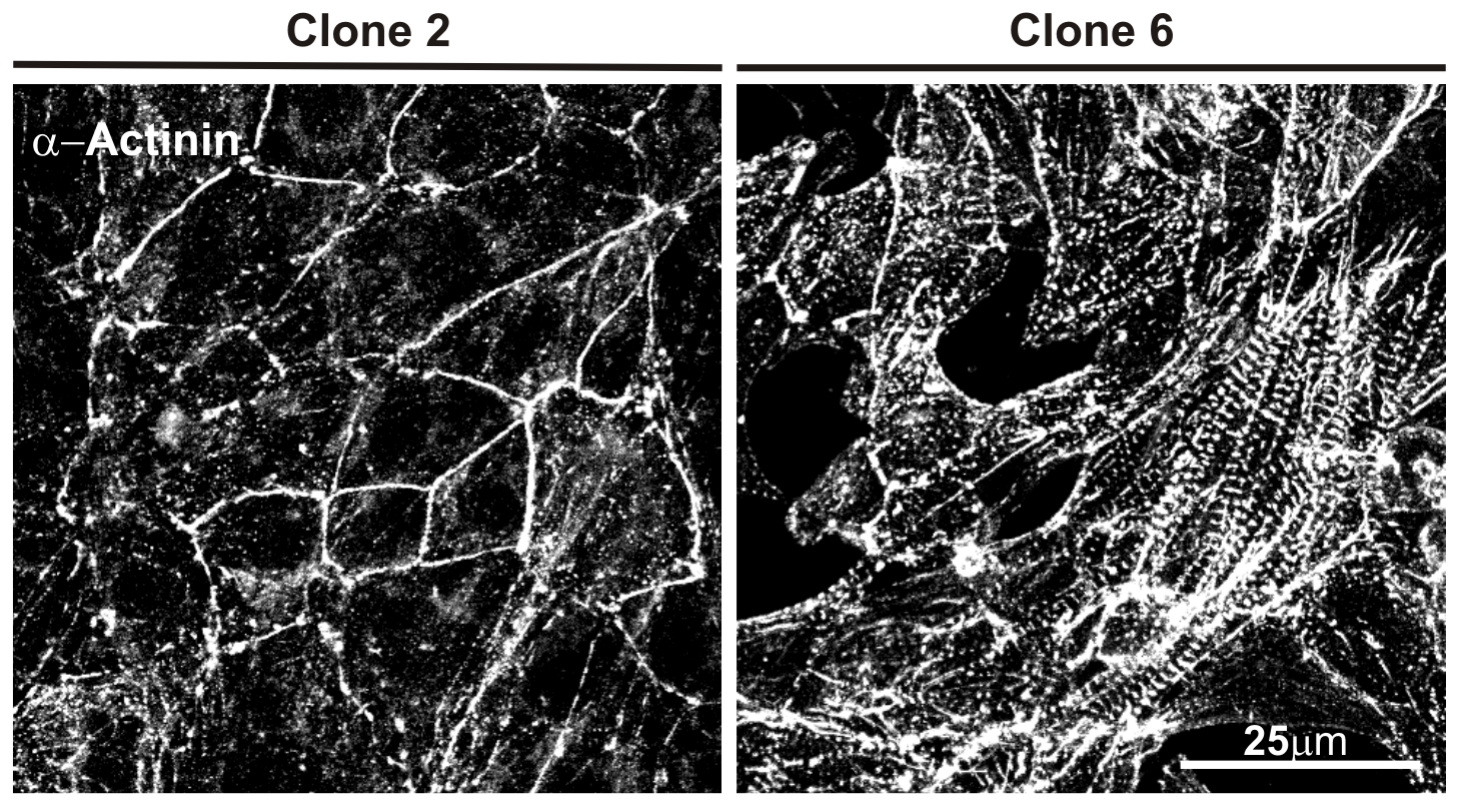
Immunofluorescent labelling of α-actinin in the HL-1 clones. Cells for clones 6 display the classic sarcomeric organisation in comparison to clone 2, where the α-actinin is associated with the cell membrane.

Cardiac specific cytoskeletal protein α-actinin located at the Z-discs of the sarcomeric assembly was highly expressed in all HL-1 clones as expected of cells isolated from a myocytic cell line (Figure 3). Clone 2 did not display the typical sarcomeric organisation of α-actinin and the protein was abnormally associated with the cell membrane.

### Immunoconfocal analysis of Cx40, Cx43 and Cx45

**Figure 4:**
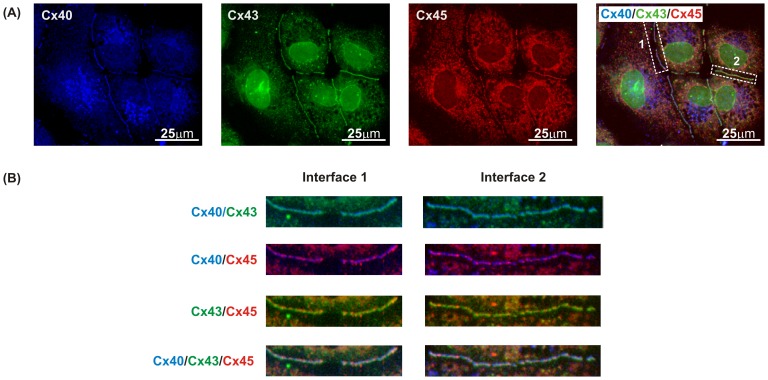
Co-localisation of Cx40, Cx43 and Cx45 in clone 6. (A) Each connexin can be seen in the individual channels within the same area and the triple labelling image was generated by superimposing the image for each connexin subtype. (B) To demonstrate the varying degrees of co-localisation at each cell interface, sections 1 and 2 highlighted from triple labelling were separated into dual images of Cx40/Cx43, Cx40/Cx45, Cx43/Cx45 and triple image of Cx40/Cx43/Cx45 as indicated on the left panel of the image.

Immunofluorescence experiments using confocal microscopy showed that all clones formed gap junctions composed of Cx40, Cx43 and Cx45. Triple labelling of Cx40, Cx43 and Cx45 in clone 6 was used to examine their relative co-localisation (Figure 4). The triple labelling image was generated by superimposing the images for each connexin subtype. This indicated that, at specific cell interfaces, all three connexins co-localised to form gap junctions, but the relative amount of signal for each connexin varied substantially from one cell interface to the next (Figure 4 (A)).

To demonstrate the varying degrees of co-localisation, two different cell interfaces from the triple labelling were separated to allow simultaneous visualisation of Cx40 and Cx43, Cx40 and Cx45 and Cx43 and Cx45 (Figure 4 (B)). Labelling for Cx40 and Cx43 gave one colour for interfaces between single cells with some interfaces predominantly showing Cx40 label while others largely showed labelling for Cx43 or a mixture of the two. Labelling for Cx40 and Cx45 showed isolated spots of Cx40 only (blue), Cx45 only (red) and a mixture of the two (purple) at different cell interfaces and within individual interfaces. A similar pattern was also observed for Cx43 (green) and Cx45 (red) with some areas of overlap of the two signals (yellow). In contrast to the homogenous colour produced by Cx40 and Cx43 at specific interfaces, Cx45 was present at variable levels and double labelling did not present any particular interface colour.

### Action potential recordings

**Figure 5:**
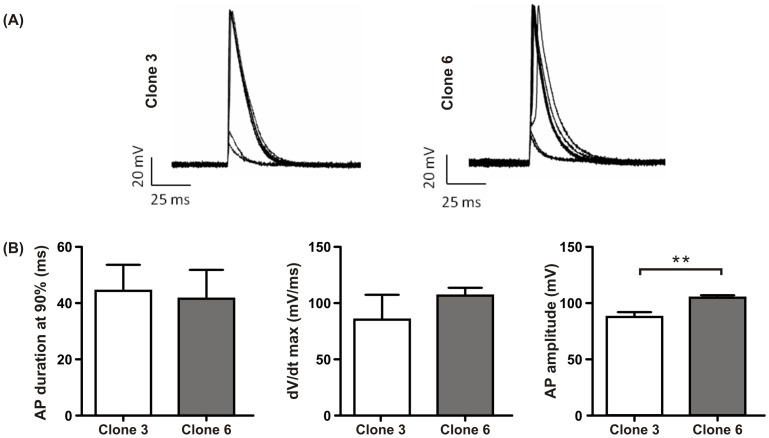
Action potential characteristics of HL-1 clones 3 and clone 6. (A) Representative action potentials recorded in clone 3 and clone 6 under whole cell current clamp mode by successive increasing depolarising pulses. (B) Quantification of (AP) amplitude, duration at 90% repolarisation (APD90) and upstroke velocity dV/dt max in clones 3 and 6.

Action potentials were recorded by applying repeated depolarising current stimulations under whole cell current clamp conditions (Figure 5). Prior to stimulation the *V*_rp_ was recorded in all three clones. We observed that clone 2 had a significantly (p<0.001) more depolarised *V*_rp_ of -46±3 mV (n = 21) when compared with clone 3 (−59±2 mV, n = 23) and clone 6 (−67±2 mV, n = 25). Action potentials in clone 2 were neither observed spontaneously or after stimulation. Action potentials were observed in clones 3 and 6 (Figure 5 (A)). We found no differences in the action potential duration measured at 90% repolarisation (clone 3: 44±8 ms, n = 7; clone 6: 42±9 ms, n = 7; p>0.05; t-test) and the dV/d*t*_max_ (clone 3: 85±18 mV/ms, n = 11; clone 6: 107±7 mV/ms, n = 11; p>0.05; t-test) (Figure 5 (B)). However the amplitude was significantly larger in clone 6 than clone 3 (clone 3: 87±4 mV, n = 7; clone 6: 105±2 mV, n = 11; p<0.005, t-test).

### Membrane currents

Voltage clamp experiments were carried out to measure the voltage gated Na^+^ (*I*_Na_) and Ca^2+^ (*I*_Ca_) currents in the HL-1 clones. Cell size (assessed by membrane capacitance) was similar in all three clones (clone 2: 13.7±0.9 pF; clone 3: 13.2±1.3 pF; clone 6: 13.7±1.4 pF; p>0.05).

**Figure 6:**
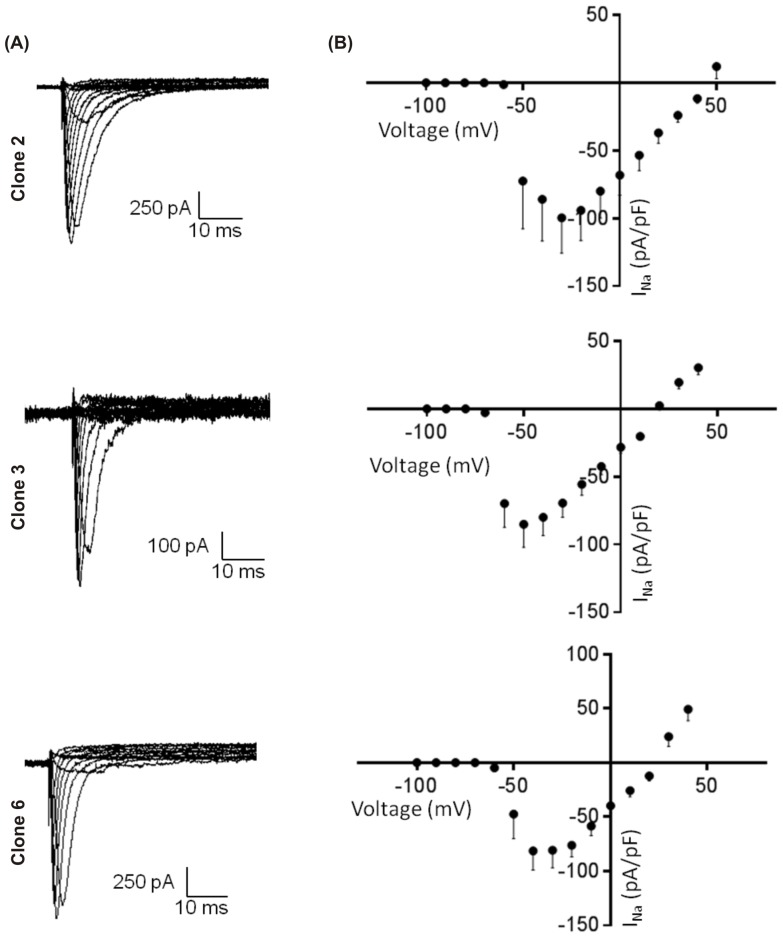
Current-voltage relationship of Na^+^ channels in HL-1 clones 2, 3 and 6. (A) Representative sodium current recorded in whole cell voltage clamp mode. (B) Corresponding averaged current-voltage relationship for each clone (B).

100% of the cells patched displayed *I*_Na_ as shown in Figure 6. There was no significant difference in the peak current density of *I*_Na_ in all the clones (clone 2: −99.5±26.3 pA/pF, n = 10; clone 3: −85.1±17.6 pA/pF, n = 11; clone 6: −81.4±17.7 pA/pF, n = 12; p>0.05). The current-voltage (I–V) relationships indicated a reversal potential (*E*_rev_) that was distinct for each clone suggesting that Na^+^ channel permeability varied and was not specific for Na^+^ (clone 2: +44.3 mV; clone 3: +19.0 mV; clone 6: +23.2 mV).

**Figure 7:**
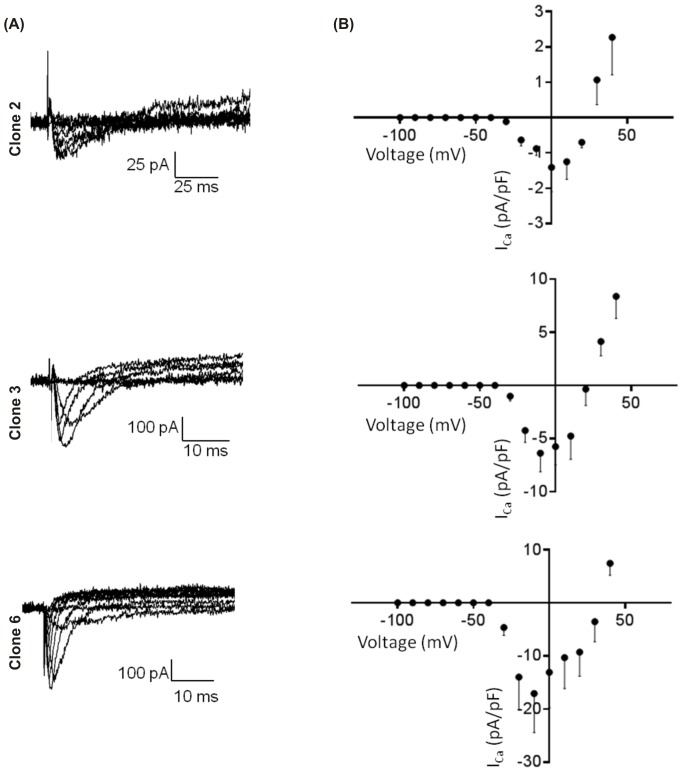
Current-voltage relationship of Ca^2+^ channels in HL-1 clones 2, 3 and 6. (A) Representative calcium current recorded in whole cell voltage clamp mode. (B) Corresponding averaged current-voltage relationship for each clone that show a specific peak current in each clone.

Contrary to *I*_Na_, *I*_Ca_ was observed in only 15% of all the cells patched (Figure 7). Clone 2 exhibited a peak current density significantly smaller than clone 3 and 6 (clone 2: −1.4±0.7 pA/pF, n = 4; clone 3: −6.4±1.9 pA/pF, n = 8; clone 6: −17.1±7.3 pA/pF, n = 7; p<0.05). Similar to *I*_Na_, the I-V relationships indicated distinct *E*_rev_ for each clone (+24.0 mV, +20.7 mV, +33.2 mV for clones 2, 3 and 6, respectively) suggesting differences in ionic permeability.

### Cell-to-cell junctional coupling

Dual voltage clamp recordings indicated no significant differences (p>0.05) in the average cell-to-cell coupling (*g*_j,0_) for each clone: clone 2: *g*_j,0_ = 14.2 ± 6.1 nS, n = 12; clone 3: *g*_j,0_ = 14.3 ± 4.6 nS, n = 8; clone 6: *g*_j,0_ = 11.2 ± 3.1 nS, n = 21.

### Calcium transient measurements

**Figure 8:**
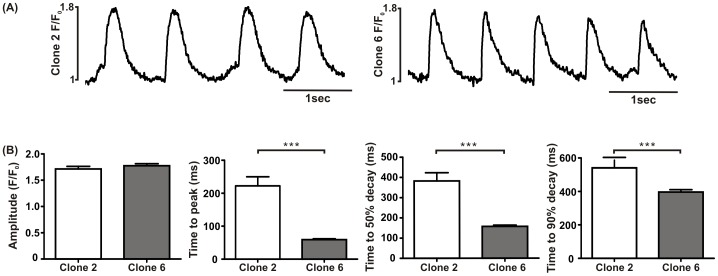
Ca^2+^ transient recordings from clones 2 and 6. (A) Spontaneous rhythmic oscillations of [Ca^2+^]_i_ in clones 2 and 6 after staining with fluo-4. (B) Both clones had a comparable [Ca^2+^]_i_ release but there was a significant prolongation in the time to peak, time to 50% decay and time to 90% decay in clone 2 compared with clone 6.

Since clones 2 and 6 displayed the most prominent differences in Ca^2+^ handling mRNA expression, Ca^2+^ transients from these cells were characterised further. For each experiment a single line scan was placed over a group of cells and the resulting Ca^2+^ transients measured for 13.3 sec (each line scan was completed in 1.33 ms and 10,000 lines were collected). Figure 8 (A) illustrates the changes in fluorescence intensity of fluo-4 measured in cells displaying spontaneous rhythmic oscillations of [Ca^2+^]_i_ from clones 2 and 6 respectively. No significant difference was found in the amplitude of [Ca^2+^]_i_ transients recorded from both clones (clone 2: 1.7±0.05 F/F_0_, n = 14; clone 6: 1.8±0.04 F/F_0_, n = 19; p>0.05; t-test). However there were significant differences in the time course of the Ca^2+^ transients between the two clones including the time to peak (clone 2: 222±27 ms, n = 5; clone 6: 59±2 ms, n = 6; p<0.0001; t-test); time to 50% relaxation (clone 2: 382±40 ms, n = 5; clone 6: 157±6 ms, n = 6; p<0.0001; t-test) and time to 90% relaxation (clone 2: 541±45 ms, n = 5; clone 6: 397±14 ms, n = 6, p = 0.002; t-test) (Figure 8 (B).

### Conduction velocity

**Figure 9:**
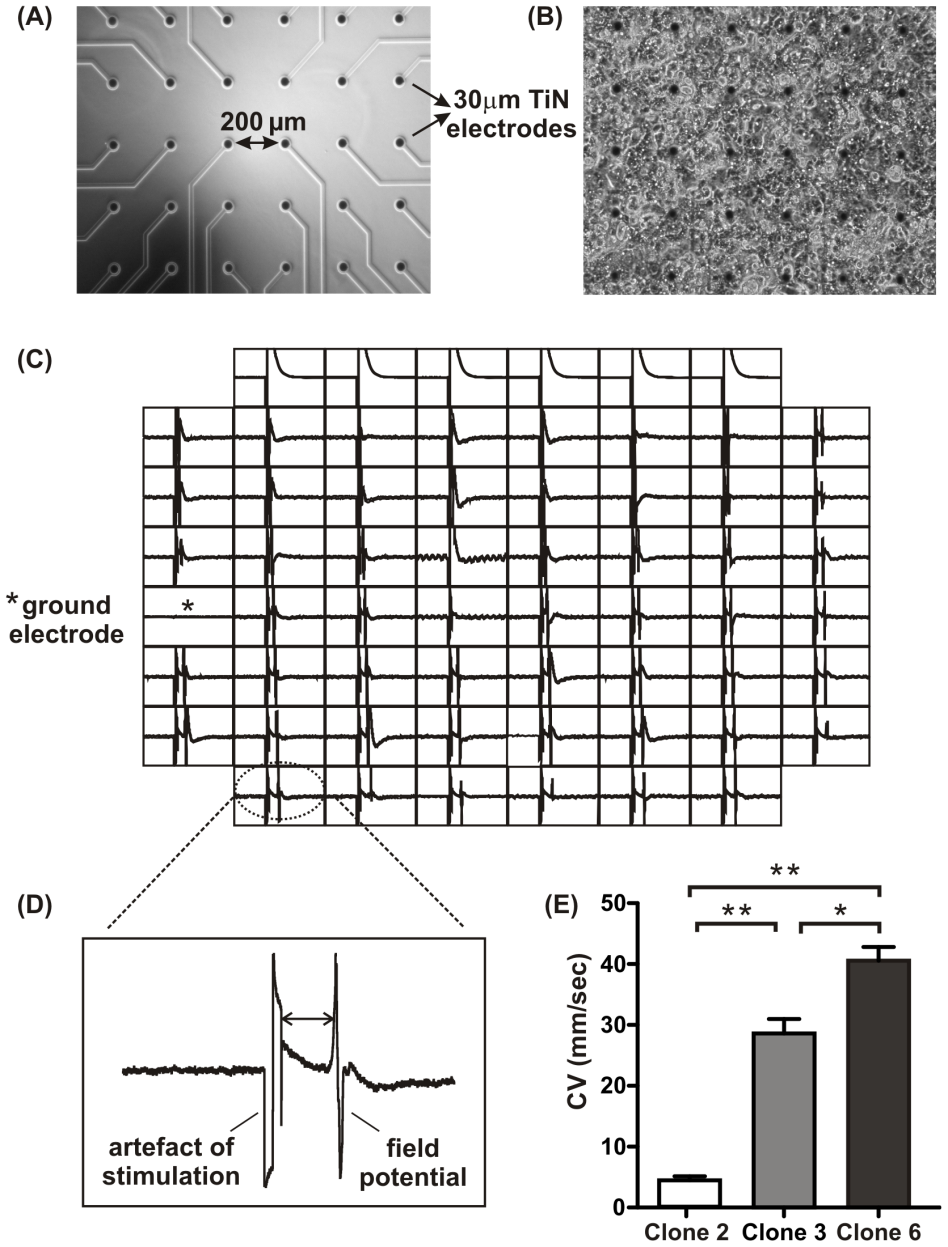
Extracellular recordings of paced cellular preparations generated from MEAs. (A) The MEA plate consists of 60 titanium nitride (TiN) electrodes. Each electrode has a diameter of 30 µM and the interelectrode distance from centre to centre is 200 µM. (B) Cells were seeded as confluent drop directly on the TiN electrodes. (C) Representative extracellular field potentials after line stimulation are shown in. (D) CV are calculated as a time delay between the artefact of stimulation and the resulting field potential against the respective distance of the electrodes from the origin of excitation in clones 2, 3 and 6 (E).

Extracellular field potentials were recorded using MEAs across confluent monolayers in clones 2, 3 and 6 (Figure 9 (A) and (B)). A spontaneous electrical activity of 1–1.5 Hz was typically observed 1–2 days post-seeding. When stimulated, the frequency of stimulation applied was always higher than the intrinsic spontaneous frequency observed to dominate the natural pacemaker. To obtain accurate CV, cells were stimulated using row stimulation from the edge of the array (top, bottom, left and right rows). Figure 9 (C) and (D) shows a typical example of an extracellular recording produced during row stimulation.

Clone 2 could not be electrically stimulated but occasional spontaneous activity was recorded which was used to estimate CV. CV in clone 2 (4±1 mm/sec; n = 6) was significantly slower (p<0.001) than clone 3 (29±2 mm/sec; n = 11) and 6 (41±1 mm/sec; n = 12) (Figure 9 (E)). CV in clones 3 and 6 were significantly different (p<0.01).

### Effects of carbenoxolone and lidocaine on conduction velocity and calcium transients

**Figure 10:**
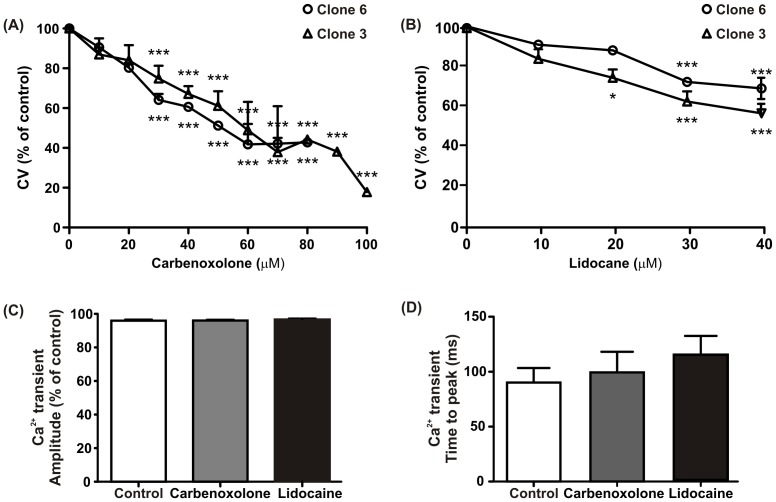
Effects of gap junctions uncoupling and the blocking of Na^+^ channels on CV and calcium transient parameters in clones 3 and 6. (A) and (B) show a dose dependent decrease in CV upon the application of carbenoxolone and lidocaine respectively in both clones. (C) and (D) There was no significant difference in Ca^2+^ transient amplitude and time to peak in the presence of carbenoxolone and lidocaine in clone 6.

Administration of carbenoxolone, a gap junction uncoupler resulted in dose-dependent reduction in CV in both clones 3 and 6 with a 50% drop in conduction achieved between 50–60 µM (clone 3: 48±14%, n = 3; clone 6: 51±1%, n = 3) (Figure 10 (A)). At concentrations above 80 µM, cells were unable to follow stimulation often resulting in conduction block particularly with clone 6. With clone 3, electrical propagation was still maintained at 100 µM with an 80% drop in CV. It should be noted that many electrodes still showed synchronous activation but because propagation was no longer linear and perpendicular to the stimulation row, velocities could no longer be measured accurately. In addition, with higher concentrations of carbenoxolone, very fast activation was often observed, most likely due to re-entry within the cellular monolayer. These fast activations could not be overcome by a higher frequency of stimulation since we observed that high pacing frequencies (above 4 Hz) reduced CV most likely because we reached a partial refractory period. Treatment with lidocaine, a Na^+^ channel blocker, had a much less pronounced effect on conduction compared with carbenoxolone; a 20–30% drop in CV was achieved using a 40 to 50 µM concentration in both clones (clone 3: 56±5%, n = 3; clone 6: 68±5%, n = 3) (Figure 10 (B)). Above these concentrations, cells were not excitable due to a large inhibition of inward Na^+^ current.

We also studied the effects of carbenoxolone (50 µM) and lidocaine (20 µM) on Ca^2+^ transients in clone 6 (Figure 10 (C) and (D)). These concentrations produced the largest changes in conduction observed by MEA. There was no significant difference (p<0.05, t-test) in amplitude (control: 95±0.77%, n = 14; carbenoxolone: 96±0.47%, n = 13; lidocaine: 96±0.60%, n = 15) and time to peak (control: 90±11 ms, n = 14; carbenoxolone: 99±16 ms, n = 13; lidocaine: 114±14 ms, n = 15) with both drugs.

## Discussion

The purpose of this work was to (1) isolate clones from low-density cultures of the original HL-1 cell line to obtain more homogenous and stable cell lines and (2) characterise these newly generated clones ensuring they displayed phenotypic characteristics consistent with cardiac cells.

At the time of sub-cloning, all clones were isolated from visually contracting colonies. After several passages, clone 2 lost the ability to contract but MEA recordings showed that spontaneous electrical activity was present. In this clone, the sarcomeric banded organisation of α-actinin was replaced by a sub-membranous distribution. Reduced contractile function due to morphological alterations in cytoskeletal proteins such as actin and titin have been reported in studies on heart failure [17]. It is likely that these differences in the sarcomeric skeleton may contribute to the loss of contractility in clone 2 [23],[46].

Comparisons of the amount of mRNA between the different clones show that only cells from clone 2 display major differences with the other clones. When clone 2 was compared with clone 6, a highly contractile clone, there was no difference in the Ca^2+^ transient amplitudes. However there was a significant prolongation in time to peak and time to decay at both 50% and 90% in clone 2 compared with clone 6. The longer time to peak in clone 2 may be explained by the significantly lower levels of L-type Ca^2+^ channel mRNA. We also consistently observed a lower Ca^2+^ peak current density in clone 2 compared with clone 6. This lower Ca^2+^ current density could lead to slower Ca^2+^ induced Ca^2+^ release. The slower decay of the Ca^2+^ transient could also be explained by lower amounts of ion transporters involved in removal of cytoplasmic Ca^2+^, namely NCX and SERCA.

Cells from clones 3 and 6 had resting membrane potentials comparable to those recorded from cells of atrial origin [35]. Despite the presence of Ca^2+^ transients and extracellular field potentials in clone 2, we did not observe action potentials in clamped cells. Cells from clone 2 also exhibited more depolarised resting membrane potentials similar to values observed in nodal cells [9]. Over this voltage range the Na^+^ channels are likely to be in an inactivated state [16]. These observations could also be explained by the large differences in cell density and time in culture after dissociation between experiments. For example, the MEAs and Ca^2+^ transients were recorded from large cellular aggregates with a minimum of three days in culture whereas patch clamp recordings required single cells and cell pairs thereby requiring a lower cell density and a much shorter time in culture. These factors of cell density and time in culture appear crucial to the differentiation levels of the HL-1 cell line exemplified by the appearance of spontaneous contractions only occurring after days in culture [4],[20],[45].

The shapes of the action potential observed in clones 3 and 6 were comparable to those obtained in cells from the original HL-1 cell line [32] and of mouse neonatal [3] (T Desplantez, personal observation) and adult atrial cells [21]. However, the action potential overshoot was higher than those recorded in the original HL-1 [32] and HL-5 cells also derived from the AT-1 cell line [43]. Action potential durations at 90% repolarisation were similar in both clone 3 and 6 but shorter than the original HL-1 cell line [32] and the HL-5 cells [43]. One would expect all three cell lines to have similar action potential characteristics as they were originally derived from the AT-1 cell line [4],[14],[43]. This illustrates the heterogeneity of the original cell lines and highlights the importance of sub-cloning enabling a more accurate analysis of the experimental results.

In clones 2, 3 and 6 recordings of *I*_Na_ currents were comparable in their peak current density but displayed different reversal potentials. In comparison to studies on adult atrial myocytes and neonatal mouse hearts, the HL-1 clones displayed maximum activation ranges at more positive potentials, especially clones 3 and 6 [13],[25],[27]. Similarly, clone 2 displays a more depolarised *E*_rev_ than clone 3, clone 6 and neonatal mouse cardiomyocytes [25]. It is possible that splice variants of the normal cardiac SCN5A gene encoding Na_v1.5_ may exist in the HL-1 clones that differ in protein synthesis, assembly and post-translation modifications producing some subtle alterations in their function [18] and their ionic permeability. It is also possible that the HL-1 clones are comparable to an embryonic phenotype unable to express the Na^+^ channels characteristic of adult cardiac myocytes [4].

Northern and western analysis confirmed that the HL-1 clones expressed a combination of Cx40 and Cx43. Cells from clone 2 displayed major differences from the other clones with lower levels of Cx40 and Cx43 for both mRNA and protein expression. The connexin expression pattern is similar to that observed in normal mouse atria [5] indicating a normal connexin expression in our HL-1 clones. Cx45 could only be detected by northern blot. Based on the exposure times required to obtain signal intensities similar to that of Cx40 and Cx43, levels of Cx45 messenger were considerably lower in all the HL-1 clones as reported *in vivo* [40]. From the quantification system developed in our laboratory that uses tagged HeLa transfectants with known amounts of connexin we estimated that Cx45 protein was expressed at least twenty times lower than Cx40 and Cx43 [33]. We also found a positive correlation between the level of mRNA and corresponding protein levels suggesting that connexin expression was predominantly regulated at the transcriptional levels similar to reports studying human myocardium [10],[40]. Despite comparable levels of Cx40, Cx43 and Cx45 expression in the HL-1 clones with mouse atria, the level of junctional coupling was eight times smaller [8]. Since the HL-1 cells are originally a cancer cell line, they may not make the necessary post-translational modifications to ensure functional junctional channels [28] [38].

Immunoconfocal analysis showed that all clones display the typical punctate gap junction labelling for the three connexins expressed. Cx45 was relatively homogenous in all clones while Cx40 and Cx43 are poorly expressed in clone 2 but more widespread in the others (data not shown). After triple labelling, a clear difference was observed in the distribution of each connexin for each individual cell-cell interface, as indicated by the resulting colours. This observation suggests two possibilities: First, a large proportion of heteromers are produced containing different ratios of Cx40, Cx43 or Cx45 with limited compatibility with other connexons. Secondly, if a large proportion of homomers form, then the known low compatibility between Cx40 and Cx43 [11],[31] would also produce this specific connexin distribution because the connexon content of each interface would depend on the level of expression of individual connexins by these two cells modified by the docking of compatible connexons at other cell-cell interfaces.

To assess CV, cells were seeded directly on top of the MEA and the extracellular field potentials were recorded upon electrical stimulation with the exception of clone 2 that could not be stimulated. Linear stimulation ensured a flat activation wavefront to avoid curvature that would lead to an underestimated CV [12]. Like neonatal ventricular myocytes in culture, propagation was not anisotropic since the cells are not elongated and the gap junctions are distributed along the cell perimeter [36]. The natural pacemaker was never observed on the array but always originated from the edge of the monolayer formed by the initial drop seeding. This is in agreement with computer simulation models since cells in the middle of the array are in a large sink (in contact with many cells) while cells on the edge have fewer intercellular contacts and therefore the current source is better matched to the load [24].

When comparing the maximum CV in the HL-1 clones, clone 2 had a significantly lower CV compared with the other clones despite the observation from patch clamp experiments showing that all the clones display no difference in *I*_Na_ current or junctional coupling. Again this may be caused by the difference in cell density and time in culture in different experimental settings. Measurements of CV in the original HL-1 cell line was 20 mm/sec, approximately half the value of clone 6 [26]. This could be caused by cellular heterogeneity as shown by Beauchamp et al. [2] using mixtures of myocytes isolated from wild type and Cx43 knock-out mice. The CV measured in the HL-1 clones was approximately ten times slower compared with mouse atrial myocytes [3]. This is most likely caused by the large difference in gap-junctional conductance which is approximately ten times smaller in the HL-1 clones and the *I*_Na_ current density which is three times smaller compared with the atrial myocytes [2]. The slow CV exhibited by the HL-1 clones permits the use of slow acquisition systems to obtain accurate measurements.

Carbenoxolone, a gap junction uncoupler, resulted in at least a 60 to 80% reduction in conduction before block occurred and/or CV was no longer measurable. The fall in CV is likely due to a decrease in intercellular coupling only as electrophysiological studies on isolated myocytes have shown that carbenoxolone has no effect on *I*_Na_ or *I*_Ca_ [6],[22]. This observation fits with the Luo-Rudy computer simulation model that predicts that reduced gap-junctional coupling causes a much larger reduction in CV (200-fold decrease before complete conduction block occurred) compared with Na^+^ channel blockade [24],[34]. This is explained by the fact that uncoupled cells will produce depolarising current flowing to fewer adjacent cells so propagation proceeds but with longer intercellular delays. Treatment with lidocaine caused a modest 20–30% conduction delay comparable to results obtained with original HL-1 cells [15]. These observations are also in keeping with the Luo-Rudy simulation of a reduction in Na^+^ conductance producing a modest drop in CV. A moderate decrease in Na^+^ channel availability still provides enough current to reach the excitation threshold [34]. On the other hand, when a large reduction of Na^+^ current occurs there is not enough junctional current to depolarise the membrane to the excitation threshold of the next connected cell(s). The voltage operated Ca^2+^ channels did not seem to play any role in these changes of CV since the Ca^2+^ transients did not show any changes with either drug indicating that the Ca^2+^ induced Ca^2+^ release mechanism was unaffected and that the voltage operated Ca^2+^ channels where therefore functioning normally. Furthermore, treatment with nifedipine abolished the Ca^2+^ transient even though it did not result in any change in CV (data not shown).

We have isolated clones of the original cell line that display very stable and homogenous phenotypes. It was essential to create a more homogenous population of cells to relate experimental data to any changes in gene/protein expression and exclude differences due to heterogeneity of the original cell line. Clone 6 displays very similar features to atrial myocytes. As such it provides a stable model for studies of conduction in atrial tissue and of components of the excitation and excitation-contraction coupling processes. The cells can be genetically manipulated (e.g. siRNA transfection) and so can provide a substrate for experiments involving the manipulation of key proteins implicated in the above functions or enable pharmacological screening.
